# Long-term safety and efficacy of daclizumab beta in relapsing–remitting multiple sclerosis: 6-year results from the SELECTED open-label extension study

**DOI:** 10.1007/s00415-020-09835-y

**Published:** 2020-05-25

**Authors:** Ralf Gold, Ernst-Wilhelm Radue, Gavin Giovannoni, Krzysztof Selmaj, Eva Kubala Havrdova, Xavier Montalban, Dusan Stefoski, Till Sprenger, Randy R. Robinson, Sami Fam, Jonathan Smith, Spyros Chalkias, Giorgio Giannattasio, Gabriel Lima, Wanda Castro-Borrero

**Affiliations:** 1 St. Josef-Hospital/Ruhr-University Bochum, 44791 Bochum, Germany; 2 Medical Image Analysis Center, University Hospital Basel, Basel, Switzerland; 3 Barts and The London School of Medicine and Dentistry, Queen Mary University of London, London, UK; 4 Department of Neurology, University of Warmia and Mazury, Olsztyn, Poland; 5 Department of Neurology, First Faculty of Medicine, Charles University, Prague, Czech Republic; 6 Hospital Vall d’Hebron University, Barcelona, Spain; 7 Rush University Medical Center, Chicago, IL USA; 8 DKD Helios Klinik Wiesbaden, Wiesbaden, Germany; 9 AbbVie Inc., Redwood City, CA USA; 10 Biogen, Cambridge, MA USA; 11 Biogen, Maidenhead, UK

**Keywords:** Daclizumab beta, Relapsing–remitting multiple sclerosis, SELECTED, Clinical trial

## Abstract

**Objective:**

SELECTED, an open-label extension study, evaluated daclizumab beta treatment for up to 6 years in participants with relapsing multiple sclerosis who completed the randomized SELECT/SELECTION studies. We report final results of SELECTED.

**Methods:**

Eligible participants who completed 1–2 years of daclizumab beta treatment in SELECT/SELECTION received daclizumab beta 150 mg subcutaneously every 4 weeks for up to 6 years in SELECTED. Safety assessments were evaluated for the SELECTED treatment period; efficacy data were evaluated from first dose of daclizumab beta in SELECT/SELECTION.

**Results:**

Ninety percent (410/455) of participants who completed treatment in SELECTION enrolled in SELECTED. Within SELECTED, 69% of participants received daclizumab beta for > 3 years, 39% for > 4 years, and 9% for > 5 years; 87% of participants experienced an adverse event and 26% a serious adverse event (excluding multiple sclerosis relapse). No deaths occurred. Overall, hepatic events were reported in 25% of participants; serious hepatic events in 2%. There were no confirmed cases of immune-mediated encephalitis. Based on weeks from the first daclizumab beta dose in SELECT/SELECTION, adjusted annualized relapse rate (95% confidence interval) for weeks 0–24 was 0.21 (0.16–0.29) and remained low on continued treatment. Overall incidence of 24-week confirmed disability progression was 17.4%. Mean numbers of new/newly enlarging T2 hyperintense lesions remained low; percentage change in whole brain volume decreased over time.

**Conclusions:**

The effects of daclizumab beta on clinical and radiologic outcomes were sustained for up to ~ 8 years of treatment. No new safety concerns were identified in SELECTED.

**Trial registration:**

Clinicaltrials.gov NCT01051349; first registered on January 15, 2010.

**Electronic supplementary material:**

The online version of this article (10.1007/s00415-020-09835-y) contains supplementary material, which is available to authorized users.

## Introduction

Relapsing–remitting multiple sclerosis (RRMS) is a neurodegenerative demyelinating disease characterized by acute relapses of neurologic symptoms and progressive accumulation of irreversible disability [4]. The goal of multiple sclerosis (MS) disease-modifying therapies (DMTs) is to reduce the frequency of MS relapses and slow the accumulation of disability [18]. Effective long-term control of MS disease activity in patients with RRMS is critical for preserving function over time [11]. Some adverse events (AEs) may only emerge after prolonged courses of treatment. Thus, it is important to evaluate the efficacy and safety of DMTs over treatment periods longer than those feasible in the initial pivotal studies.

Daclizumab beta is a humanized monoclonal antibody that binds the CD25 subunit of the high-affinity interleukin-2 (IL-2) receptor and reversibly modulates IL-2 signaling [2]. Blockade of CD25 by daclizumab beta prevents formation of the high-affinity IL-2 receptor and shifts IL-2 signaling to the intermediate-affinity receptor [2, 24]. This results in expansion of CD56^bright^ natural killer cells, antagonism of proinflammatory T cells, and reduction in the number of regulatory T cells [2, 24, 26, 30]. Daclizumab beta was approved in the United States, Europe, Canada, and other countries for the treatment of relapsing forms of MS based on evidence from pivotal clinical trials that showed superior efficacy vs placebo and intramuscular interferon beta-1a on key clinical and radiologic disease outcomes [2, 15, 21] and a favorable risk–benefit profile at the time of marketing authorization (May and July 2016 in the United States and Europe, respectively) [9]. In the United States, daclizumab was available through a restricted distribution program due to serious safety risks, including liver injury and immune conditions [29]. Daclizumab beta was formulated to be self-administered subcutaneously (SC) once monthly [2].

**Fig. 1:**
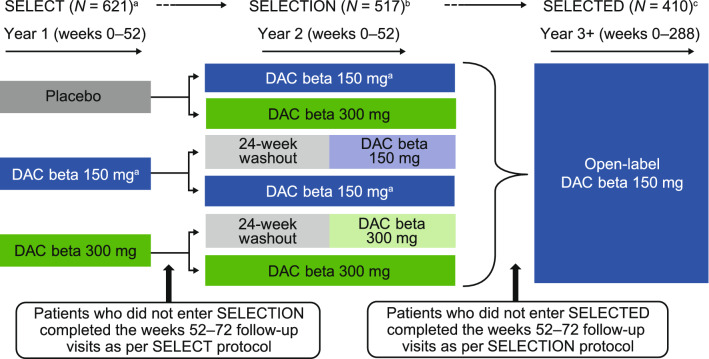
SELECT trilogy study design. Daclizumab beta or placebo was administered subcutaneously every 4 weeks in each of the studies. ^a^Gold et al. [15]. ^b^Giovannoni et al. [12]. ^c^Gold et al. [16]. Figure reproduced from Gold et al. [16] distributed under the terms of the Creative Commons Attribution 4.0 International License (https://creativecommons.org/licenses/by/4.0/) with the following revisions: generic name updated to daclizumab beta

SELECTED (NCT01051349) was the third and final study in the SELECT trilogy of studies (Fig. 1). SELECTED was an open-label, single-arm study that evaluated extended open-label treatment with daclizumab beta 150 mg every 4 weeks in participants who completed both SELECT [15] and SELECTION [12]. An interim analysis of SELECTED (data collected as of January 2014) showed that continuous treatment with daclizumab beta was effective over 3 years of treatment in participants with RRMS [16]. The yearly incidence of AEs did not increase over time, and the safety profile was consistent with previous observations from SELECT and SELECTION [16]. SELECTED was designed to follow participants who were receiving open-label daclizumab beta 150 mg SC every 4 weeks for up to 6 years after completion of SELECTION.

In June 2017, a review of daclizumab was initiated by the European Medicines Agency (EMA) Pharmacovigilance Risk Assessment Committee (PRAC) following a fatal case of fulminant liver failure [10], in addition to four cases of serious liver injury. Based on PRAC recommendations, daclizumab beta was restricted to the treatment of patients with highly active disease despite a full and adequate course of treatment with at least one DMT, or with rapidly evolving severe RMS who are unsuitable for treatment with other DMTs, and required additional monitoring to minimize the risk of serious liver injury [6, 10]. On March 2, 2018, following cases of inflammatory encephalitis and meningoencephalitis, the PRAC initiated an Article 20 referral procedure for daclizumab beta [8]. On the same day, Biogen and AbbVie announced their decision to voluntarily withdraw the worldwide marketing authorizations for daclizumab beta, given the limited number of patients being treated and the evolving benefit/risk profile [1, 28]. On March 7, 2018, the EMA recommended suspending the marketing authorization of daclizumab beta in the European Union and recalling the product [7, 28].

Despite the fact that daclizumab beta is no longer a marketed product for the treatment of RRMS, it is important for the safety and efficacy data to be in the public domain, to provide additional context on daclizumab beta effects in RRMS. This report provides the final safety and efficacy results from SELECTED after up to 8 years of daclizumab treatment.

## Methods

### Study design

The SELECT trilogy study design has been described previously (Fig. 1) [16]. Participants who completed SELECT and SELECTION were eligible for inclusion in SELECTED, an international, single-arm, open-label extension study evaluating 6 years of treatment with daclizumab beta 150 mg SC every 4 weeks. SELECTED was conducted across 66 study centers in eight countries; the first participant was enrolled/treated on March 31, 2010. The primary objective of SELECTED was to assess the safety of long-term daclizumab beta monotherapy in participants with RRMS. Efficacy assessment, including number of relapses, assessment of disability progression, number of new/newly enlarging T2 hyperintense lesions, and annualized percentage change in whole brain volume (PCBV) were secondary objectives. The SELECTED protocol was approved by central and local ethics committees and the study was conducted in accordance with the International Conference on Harmonization Guideline for Good Clinical Practice [19] and the Declaration of Helsinki.

### Study participants and procedures

Eligibility criteria for SELECT and SELECTION have been previously reported [12, 15]. Participants who completed 52 weeks of daclizumab beta treatment in SELECTION were eligible to enroll in SELECTED and receive open-label daclizumab beta 150 mg SC every 4 weeks for up to 6 more years. Participants who permanently discontinued study treatment because of an AE, had enrolled in any other investigational study, or who were receiving ongoing treatment with any other DMT for MS were ineligible to participate. Other participants ineligible to enroll in SELECTED were those currently taking valproic acid, carbamazepine, lamotrigine, or phenytoin; those receiving treatment with isoniazid, propylthiouracil, or nimesulide; and those who had any significant change in their medical status from the previous study, including laboratory tests or a clinically significant condition that, in the opinion of the investigator, precluded treatment with daclizumab beta. Women of child-bearing potential were required to use effective contraception with at least one contraceptive method during the study and up to 4 months after their last dose of daclizumab beta. Written informed consent was obtained from each participant prior to eligibility evaluation. Participants who experienced a relapse were required to re-consent at the next study visit to continue study participation.

SELECTED was terminated early to consolidate ongoing extension studies in the daclizumab beta program; participants who were still on treatment as of April 2015 were transitioned to the EXTEND (NCT01797965) long-term extension study, if eligible. EXTEND was an umbrella long-term extension study that included participants from the different ongoing trials, including the phase 3 DECIDE trial. Participants who enrolled in EXTEND were considered to have completed SELECTED. EXTEND began in February 2013. Daclizumab beta dosing subsequently ceased in March 2018. Data reported here reflect those collected within SELECTED or the SELECT trilogy.

The safety and the intention-to-treat efficacy populations consisted of all participants who received at least one dose of daclizumab beta in SELECTED.

### Safety assessment

Safety evaluation included all treatment-emergent AEs that occurred or worsened in severity from the first dose in SELECTED until up to 6 months after the last dose of daclizumab beta in SELECTED.

Safety and tolerability assessments included AE monitoring, physical and neurological exams, vital signs, electrocardiograms, and clinical lab evaluations. Investigators rated AE severity (mild, moderate, or severe) based on protocol guidance and regulatory criteria for a serious AE (SAE). An SAE was an AE that resulted in death, persistent or significant disability/incapacity, or a congenital anomaly/birth defect; required inpatient hospitalization or prolongation of existing hospitalization; or was a life-threatening event, in the opinion of the investigator. Participants who experienced a clinically significant cutaneous AE were referred to a dermatologist. Liver function testing (alanine aminotransferase [ALT], aspartate aminotransferase [AST], and total bilirubin) was performed monthly; results from a liver function test given within the previous 7 days were reviewed by a neurologist and had to be within protocol-defined limits before a dose of daclizumab beta could be administered.

### Efficacy assessment

Relapses were defined as new/recurrent neurological symptoms with new objective neurological findings (confirmed by a neurologist) lasting at least 24 h and not associated with fever/infection. Gradually evolving new/recurrent neurological symptoms (over months) were considered as disability progression, not acute relapses. New/recurrent neurological symptoms that occurred < 30 days after a protocol-defined relapse were considered part of the same relapse. Expanded Disability Status Scale (EDSS) score was assessed at baseline (defined as the most recent assessment [not occurring during relapse] prior to the first dose of daclizumab beta in SELECTED) and every 24 weeks thereafter. Participants who had a ≥ 1.0-point increase in EDSS score from a baseline EDSS score of ≥ 1.0 or a ≥ 1.5-point increase in EDSS score from a baseline EDSS score of < 1.0 were re-assessed 12 and 24 weeks later to determine if disability progression had occurred. Brain MRI scans were performed at baseline, at weeks 48, 96, 192, 240, and at the end of treatment visit (week 288), and were read at a central reading institution (Medical Image Analysis Center, Basel, Switzerland) [16]. The original protocol included brain MRI scans at week 144, but this assessment was removed in a global amendment to the protocol in May 2013.

### Efficacy endpoints

Efficacy outcomes were assessed for each participant based on time from first dose of daclizumab beta received in SELECT or SELECTION and continuing through the SELECTED study period, for up to 8 years of analyses (1 year for SELECT, 1 year for SELECTION, and up to 6 years for SELECTED). Efficacy endpoints included time to first relapse and to 24-week confirmed disability progression (CDP), annualized relapse rate (ARR) by 6-month intervals, the number of new or newly enlarging T2 hyperintense lesions by yearly intervals, and the annualized PCBV by yearly intervals. For the analysis of new or newly enlarging T2 hyperintense lesions and annualized PCBV by yearly intervals, data had to be reported for the year prior to the year being assessed.

### Statistical analyses

ARR was calculated as the total number of relapses divided by the total number of days up to the end of the study; the ratio was multiplied by 365.25. The adjusted ARR was estimated from a Poisson regression adjusted for number of relapses in the year before study entry. In addition, relapse rates for individual participants were calculated as the number of relapses for that patient divided by the number of days they participated in the study; the ratio was multiplied by 365.25. Based on these individual relapse rates, the mean and median were calculated.

The estimated time to 24-week CDP (defined as a ≥ 1.0-point increase on the EDSS from the baseline EDSS ≥ 1.0 that was sustained for 24 weeks, or a ≥ 1.5-point increase on the EDSS from baseline EDSS < 1.0 that was sustained for 24 weeks) and proportion of participants with 24-week CDP were evaluated based on Kaplan–Meier product limit method. The date of the first dose of daclizumab beta was used as the start date.

## Results

### Study participants

**Fig. 2:**
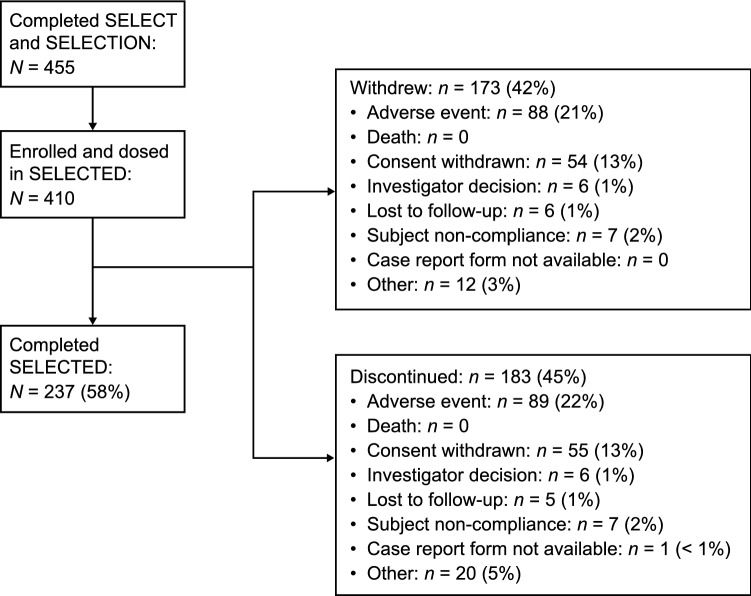
Participant disposition. Participants who completed SELECT and SELECTION were eligible to enroll in SELECTED. Reasons for withdrawal from the study and discontinuation of the study drug are shown

Of 455 participants who completed SELECT and SELECTION, 410 participants (90%) enrolled and were dosed in SELECTED (Fig. 2). Participant characteristics at baseline in SELECTED are shown in Table 1. The first participant was enrolled/treated in SELECTED on March 31, 2010, and the date of the last participant visit was August 25, 2016. Overall, 237 (58%) of participants completed the study; of these, 227 transitioned to EXTEND and were considered to have completed treatment. The number of participants who discontinued study drug each year remained stable throughout the study (10–13% per study year). The mean (median [range]) time on treatment in SELECTED was 40.2 (45.1 [0–66]) months (1460.6 patient-years of exposure). In total, 173 (42%) of participants withdrew from the study and 183 (45%) discontinued treatment; the most common reasons for treatment discontinuation were AEs (89 participants [22%]) and consent withdrawn (55 participants [13%]). The reasons/frequency for study withdrawal were similar to those for discontinuation. Within the SELECTED study, participants had received a median (range) of 49 (1–72) doses; 69% of participants received daclizumab beta for > 3 years, 39% for > 4 years, and 9% for > 5 years. Safety and efficacy populations for the analysis were the same, comprising all participants dosed in SELECTED.

**Table 1:** Participant demographics and clinical characteristics at SELECTED baseline

Characteristic	Study population (*N* = 410)
Previous treatment assignment^a^	
Placebo (SELECT); DAC 150 mg (SELECTION)	70 (17)
Placebo (SELECT); DAC 300 mg (SELECTION)	66 (16)
DAC 150 mg (SELECT); placebo/DAC 150 mg (SELECTION)	69 (17)
DAC 150 mg (SELECT and SELECTION)	69 (17)
DAC 300 mg (SELECT); placebo/DAC 300 mg (SELECTION)	71 (17)
DAC 300 mg (SELECT and SELECTION)	65 (16)
Age (years), mean (SD)	38 (9)
Female (%)	62
No. of relapses in prior study, mean (SD)^b^	0.2 (0.5)
Range	0–3
EDSS score, mean (SD)	2.7 (1.3)
Range	0–6
No. of Gd^+^ lesions, mean (SD)	0.2 (1.0)
Range	0–12
Participants with ≥ 1 Gd^+^ lesions, *n* (%)	40 (10)
No. of T2 hyperintense lesions, mean (SD)	46.3 (36.5)
Range	0–194
T2 hyperintense lesion volume, mm^3^, median	3868
T1 hypointense lesion volume, mm^3^, median	751
Months on treatment, median^c^	45.1
Range	< 1–66
Doses, mean (SD)	44.0 (19.4)
Median	49.0
Range	1–72

*DAC* daclizumab beta, *EDSS* Expanded Disability Status Scale, *Gd*^+^ gadolinium-enhancing, *SC* subcutaneous, *SD* standard deviation

^a^Participants in SELECTION who had been treated with daclizumab beta 150 mg or 300 mg in SELECT were randomized to a 6-month placebo washout followed by re-initiation of their previous dosage of daclizumab beta or to continue their daclizumab beta dosage in SELECTION

^b^Includes all relapses in SELECTION, whether in the treatment period, randomized washout phase, or follow-up period, either confirmed or not confirmed by an independent neurology evaluation committee

^c^Time on treatment, in days, was derived as (date of last dose) − (date of first dose in SELECTED) + 1

Table adapted from Gold et al. [16] distributed under the terms of the Creative Commons Attribution 4.0 International License (https://creativecommons.org/licenses/by/4.0/) with the following revisions: data added for previous treatment assignment

### Safety overview and incidence of AEs and SAEs

AEs were summarized by study year (weeks 1–48, weeks 49–96, weeks 97–144, weeks 145–192, and week 193 and above) and overall. Year on year, the incidence of AEs, SAEs, and AEs resulting in discontinuation did not increase over time and no deaths were reported in SELECTED (Table 2). Overall, 87% of participants experienced an AE (20% mild, 54% moderate, and 13% severe), 26% had SAEs (excluding MS relapse), 22% had AEs leading to treatment discontinuation (Table 3), and 40% had an AE reported to be related to the study drug. Common AEs occurring in 10% of participants or more were MS relapse (29%), nasopharyngitis (17%), upper respiratory tract infection (15%), increased ALT (15%), increased AST (12%), headache (11%), urinary tract infection (11%), pharyngitis (10%), and back pain (10%; a listing of AEs with a > 5% incidence are shown in Table 4). The most frequently reported SAEs, excluding MS relapse, were urinary tract infection and lymphadenopathy [in five and six participants, respectively; 1% of participants for both (Table 5)].

**Table 2:** Summary of AEs by yearly intervals and overall during the SELECTED treatment period

Adverse event, *n* (%)	Daclizumab beta 150 mg SC					
	Weeks 1–48 (*n* = 410)	Weeks 49–96 (*n* = 387)	Weeks 97–144 (*n* = 343)	Weeks 145–192 (*n* = 307)	Week 193 and above (*n* = 250)	Overall (*n* = 410)
All AEs	246 (60)	233 (60)	196 (57)	172 (56)	127 (51)	358 (87)
Severity of AEs						
Mild	120 (29)	103 (27)	79 (23)	79 (26)	54 (22)	84 (20)
Moderate	113 (28)	118 (30)	104 (30)	82 (27)	63 (25)	222 (54)
Severe	13 (3)	12 (3)	13 (4)	11 (4)	10 (4)	52 (13)
All SAEs	53 (13)	47 (12)	42 (12)	38 (12)	34 (14)	148 (36)
SAEs excluding MS relapse	23 (6)	26 (7)	27 (8)	22 (7)	24 (10)	105 (26)
AEs leading to treatment discontinuation	22 (5)	19 (5)	13 (4)	16 (5)	21 (8)	91 (22)
Death	0	0	0	0	0	0

*AE* adverse event, *MS* multiple sclerosis, *SAE* serious adverse event, *SC* subcutaneous

**Table 3:** AEs leading to treatment discontinuation in three or more participants by MedDRA SOC and PT during the SELECTED treatment period

AE, *n* (%)	Daclizumab beta 150 mg SC (*N* = 410)
Any AE leading to treatment discontinuation	91 (22)
Skin and subcutaneous tissue disorders	15 (4)
Psoriasis	3 (< 1)
Investigations	27 (7)
Increased ALT	17 (4)
Increased AST	8 (2)
Increased hepatic enzyme	5 (1)
Increased GGT	4 (< 1)
Abnormal liver function test	4 (< 1)
Infections and infestations	6 (1)
Gastrointestinal disorders	10 (2)
Colitis	3 (< 1)
Hepatobiliary disorders	11 (3)
Liver disorder	4 (< 1)
Blood and lymphatic system disorders	8 (2)
Lymphadenopathy	5 (1)
Neoplasms benign, malignant, unspecified	7 (2)
Breast cancer	3 (< 1)

*AE* adverse event, *ALT* alanine transaminase, *AST* aspartate transaminase, *GGT* gamma-glutamyltransferase, *MedDRA* Medical Dictionary for Regulatory Activities, *PT* Preferred Term, *SC* subcutaneous, *SOC* System Organ Class

**Table 4:** Common AEs (occurring in 5% of participants or more) during the SELECTED treatment period^a^

AE, *n* (%)	Daclizumab beta 150 mg SC (*N* = 410)
MS relapse	119 (29)
Nasopharyngitis	68 (17)
Upper respiratory tract infection	61 (15)
Increased ALT	61 (15)
Increased AST	49 (12)
Headache	44 (11)
Urinary tract infection	44 (11)
Pharyngitis	42 (10)
Back pain	40 (10)
Respiratory tract infection (viral)	35 (9)
Allergic dermatitis	32 (8)
Rash	31 (8)
Bronchitis	29 (7)
Diarrhea	25 (6)
Lymphadenopathy	25 (6)
Viral infection	23 (6)
Arthralgia	22 (5)
Fall	21 (5)
Eczema	20 (5)
Increased GGT	20 (5)
Respiratory tract infection	20 (5)
Oral herpes	20 (5)
Rhinitis	20 (5)

*AE* adverse event, *ALT* alanine aminotransferase, *AST* aspartate aminotransferase, *GGT* gamma-glutamyltransferase, *MS* multiple sclerosis, *SC* subcutaneous

^a^AEs in ≥ 5% of participants by Medical Dictionary for Regulatory Activities Preferred Term. Participants were counted only once within each Preferred Term

**Table 5:** SAEs occurring in two or more participants during the SELECTED treatment period^a^

SAE, *n* (%)	Daclizumab beta 150 mg SC (*N* = 410)
Any SAE	148 (36)
MS relapse	62 (15)
Lymphadenopathy	6 (1)
Urinary tract infection	5 (1)
Breast cancer	3 (< 1)
Ulcerative colitis	3 (< 1)
Toxic skin eruption	3 (< 1)
Urticaria	3 (< 1)
Autoimmune hepatitis	2 (< 1)
Bronchitis	2 (< 1)
Cholelithiasis	2 (< 1)
Concussion	2 (< 1)
Dermatitis allergic	2 (< 1)
Foot fracture	2 (< 1)
Gastritis	2 (< 1)
Herpes zoster	2 (< 1)
Increased hepatic enzyme	2 (< 1)
Intervertebral disc disorder	2 (< 1)
Lower limb fracture	2 (< 1)
Lymphadenitis	2 (< 1)
Pneumonia	2 (< 1)
Road traffic accident	2 (< 1)
Upper limb fracture	2 (< 1)
Wound infection	2 (< 1)

*MS* multiple sclerosis, *SAE* serious adverse event, *SC* subcutaneous

^a^SAEs in ≥ 2 patients by Medical Dictionary for Regulatory Activities Preferred Term

#### Hepatic events

Drug-related hepatic AEs were reported in 25%, with serious hepatic AEs in 2% (Table 6). Incidence of ALT and AST elevations were collected as AEs. Overall, the incidence of ALT or AST elevations ≥ 3 × upper limit of normal (ULN) was 15%, and the incidence of ALT and AST elevations > 5 × ULN was 9% and 6%, respectively; 4% of participants had ALT or AST levels > 10 × ULN. Two participants had liver transaminase elevations ≥ 3 × ULN with concurrent elevation of bilirubin values > 2 × ULN within 3 months of study drug discontinuation. Both cases were medically assessed to be confounded by other factors: specifically, one study participant experienced toxic liver disease considered by the investigator to be secondary to valproate treatment approximately 2.5 months after discontinuing study treatment. At the time of this event, the individual had received 67 doses of daclizumab beta over 5 years and 2 months of participation in the SELECT trilogy. The second participant experienced hepatocellular jaundice with elevated liver function tests approximately 8 weeks after study treatment discontinuation, following treatment of a cutaneous AE (toxic skin eruption) with herbal supplements and having taken influenza medication containing paracetamol. Two additional participants had ALT/AST > 3 × ULN with concurrent bilirubin > 2 × ULN at laboratory assessments during hospitalizations that were not captured in the clinical database because they occurred outside of a clinical study site; these cases were medically reviewed. The independent hepatic adjudication committee concluded that none of the four cases met the criteria for Hy’s law. However, three of the four cases were considered by the hepatic adjudication committee to be possibly related to study drug.

**Table 6:** Adverse events of special interest from first dose of daclizumab beta in SELECTED

Adverse event, *n* (%)	Total (*N* = 410)
Hepatic events^a^	
AE	104 (25)
SAE	10 (2)
Hepatic enzyme increase	2 (< 1)
Autoimmune hepatitis	2 (< 1)
Hepatitis	1 (< 1)
Liver function test abnormal	1 (< 1)
Liver disorder	1 (< 1)
Hepatocellular jaundice	1 (< 1)
Alanine aminotransferase increased	1 (< 1)
Aspartate aminotransferase increased	1 (< 1)
Hepatic laboratory abnormalities^b^	
ALT or AST ≥ 3 × ULN	60 (15)
ALT or AST > 5 × ULN	37 (9)
ALT or AST > 10 × ULN	16 (4)
ALT or AST ≥ 3 × ULN and concurrent bilirubin > 2 × ULN	2 (< 1)^c^
Hy's law	0
Cutaneous events^d^	
AEs	157 (38)
SAEs	18 (4)
Urticaria	3 (< 1)
Toxic skin eruption	3 (< 1)
Allergic dermatitis	2 (< 1)
Psoriasis	2 (< 1)
Erythrodermic psoriasis	1 (< 1)
Photodermatitis	1 (< 1)
Angioedema	1 (< 1)
Dermatitis	1 (< 1)
Drug eruption	1 (< 1)
Eczema	1 (< 1)
Erythema nodosum	1 (< 1)
Seborrheic dermatitis	1 (< 1)
Stevens–Johnson syndrome^e^	1 (< 1)
Infections^f^	
AE	252 (61)
SAE	23 (6)
Urinary tract infection	5 (1)
Pneumonia	2 (< 1)
Bronchitis	2 (< 1)
Herpes zoster	2 (< 1)
Wound infection	2 (< 1)
Gastrointestinal infection	1 (< 1)
Hepatitis C	1 (< 1)
Infectious mononucleosis	1 (< 1)
Upper respiratory tract infection	1 (< 1)
Acute sinusitis	1 (< 1)
Appendicitis	1 (< 1)
Clostridium difficile colitis	1 (< 1)
Diverticulitis	1 (< 1)
Erysipelas	1 (< 1)
Furuncle	1 (< 1)
HIV infection	1 (< 1)
Peritonsillar abscess	1 (< 1)
Pyelonephritis acute	1 (< 1)
Tonsillitis	1 (< 1)
Lymphadenopathy events^g^	
AE	28 (7)
SAE	9 (2)
Lymphadenopathy	6 (1)
Lymphadenitis	2 (< 1)
Lymphoid tissue hyperplasia	1 (< 1)
Depression and suicidal ideation^h^	
AE	24 (6)
SAE	1 (< 1)
Depression and suicide attempt	1 (< 1)
Inflammatory gastrointestinal event^i^	
AE	11 (3)
SAE	6 (1)
Ulcerative colitis	3 (< 1)
Colitis	1 (< 1)
Crohn’s disease	1 (< 1)
Hemorrhagic enterocolitis	1 (< 1)
Malignancies^j^	
AE^k^	10 (2)
SAE	7 (2)
Breast cancer	3 (< 1)
T-cell lymphoma	1 (< 1)
Anal cancer	1 (< 1)
Clear cell renal cell carcinoma	1 (< 1)
Carcinoid tumor pulmonary	1 (< 1)

*AE* adverse event, *ALT* alanine aminotransferase, *AST* aspartate aminotransferase, *HLT* high-level term, *SAE* serious AE, *SMQ* Standardized MedDRA Queries, *SOC* system organ class, *ULN* upper limit of normal

^a^Sublevel SMQ of Drug-related hepatic disorders—comprehensive search under the SMQ of Hepatic disorders

^b^Only treatment-emergent lab values are included

^c^In one participant, these elevations were noted during a hospitalization and assessed as toxic liver disease considered secondary to valproate treatment approximately 2.5 months after discontinuing study treatment. In the other case, the participant experienced hepatocellular jaundice with elevated liver function tests approximately 8 weeks after study treatment discontinuation, following treatment of a skin event with herbal supplements and having taken influenza medication containing paracetamol. Neither was assessed as related to study treatment in the opinion of the investigator. Neither case met Hy’s law criteria. Two additional participants had ALT/AST > 3 × ULN with concurrent bilirubin values > 2 × ULN at laboratory assessments during hospitalization that were not captured in the clinical database

^d^SOC of Skin and subcutaneous tissue disorders

^e^The reported case of Stevens–Johnson syndrome was adjudicated by the local dermatologist and the central independent dermatologist as not consistent with Stevens–Johnson syndrome

^f^SOC of Infections and infestations

^g^Customized MedDRA search under selected HLTs

^h^SMQ of Depression and suicide/self-injury

^i^HLT of Colitis (excluding infective), primary or secondary pathway

^j^Events in the analysis of potential malignancies confirmed upon medical review

^k^The other three non-SAE malignancies were Hodgkin’s disease, penile cancer, and basal cell carcinoma

#### Cutaneous AEs

Cutaneous events were reported in 38% of participants, with serious cutaneous AEs in 4%. Cutaneous SAEs reported in more than one participant were urticaria and toxic skin eruption (three participants each), allergic dermatitis, and psoriasis (two participants each; Table 6). One serious cutaneous AE was reported as Stevens-Johnson syndrome by the treating neurologist, but the diagnosis was not supported by the case details per the local site dermatologist and the central independent dermatologist assessments, as it was moderate in intensity, localized, lacked any bullous or necrotic skin lesions, and had no areas of skin loss, and there was full thickness of the epidermis [16].

Most cutaneous AEs were not injection site reactions. The most common cutaneous AEs were rash (8%), allergic dermatitis (8%), and eczema (5%). The yearly incidence of cutaneous events did not increase over time, and events were reported in 15% of participants during weeks 1–48, 14% during weeks 49–96, 12% during weeks 97–144, 14% during weeks 145–192, and 11% during week 193 and above. Some participants had recurrence of cutaneous AEs across different epochs. The majority of participants experienced cutaneous AEs that were mild or moderate in severity, while seven (2%) participants had cutaneous AEs that were severe. Cutaneous events led to discontinuation of study treatment in 4% of participants.

#### Infections

Infections were reported in 61% of participants. The most commonly reported AEs of infections (occurring in 10% or more of participants) were nasopharyngitis (17%), upper respiratory tract infections (15%), urinary tract infections (11%), and pharyngitis (10%). The yearly incidence of infections did not increase over time, and infections were reported in 34% of participants during weeks 1–48, 32% during weeks 49–96, 36% during weeks 97–144, 28% during weeks 145–192, and 26% during weeks 193 and above. The majority of infections were mild or moderate in severity, and 2% were severe. One percent of participants discontinued treatment due to infections.

The incidence rate of serious infections was 6%. Serious infections occurring in two or more participants included urinary tract infection, pneumonia, bronchitis, herpes zoster, and wound infection (Table 6). There were five reports of potential opportunistic infections (1%), including vulvovaginal candidiasis, oral candidiasis, fungal pneumonia, and pulmonary tuberculosis. Pulmonary tuberculosis was reported in a participant from Ukraine who had received daclizumab beta for 2.5 years (33 total doses). The investigator assessed the AE as severe and not related to daclizumab beta. Treatment was discontinued and the participant withdrew from the study. In six participants, daclizumab beta was temporarily interrupted due to serious infection: herpes zoster in two participants; and diverticulitis, wound infection, erysipelas, and pneumonia in one participant each.

#### Lymphadenopathy events

Lymphadenopathy-related events were reported in 7% of participants using a customized Medical Dictionary for Regulatory Activities (MedDRA) search for Preferred Terms under selected high-level terms. The most commonly reported lymphadenopathy-related event was lymphadenopathy (6%); all other lymphadenopathy-related Preferred Terms in the customized search were reported in ≤ 1% of participants. The majority of lymphadenopathy-related events were mild or moderate in severity. Severe lymphoid tissue hyperplasia was experienced by one participant. Lymphadenopathy-related events led to discontinuation of study treatment in 1% of participants. The incidence of lymphadenopathy-related SAEs was 2%. One SAE of lymphadenopathy and one of lymphadenitis were assessed as related to study treatment by the investigators. The overall incidence of lymphadenopathy-related events was stable over time, with no evidence of lymphoma.

#### Gastrointestinal AEs

Gastrointestinal AEs (as defined in the MedDRA System Organ Class of Gastrointestinal Disorders) were reported in 24% of participants; the majority were mild or moderate in severity. The incidence of serious gastrointestinal AEs was 3%. The only gastrointestinal event that led to discontinuation of study treatment in more than one participant was colitis (three participants). Six (1%) participants reported serious gastrointestinal events: three cases of ulcerative colitis and one case each of colitis, Crohn’s disease, and hemorrhagic enterocolitis. Treatment included discontinuation of study treatment and standard therapies for colitis, including mesalazine, sulfasalazine, corticosteroids, and azathioprine. The majority of AEs resolved or were stable with no flares following discontinuation of study treatment and/or treatment for the events. A total of 11 participants (3%) had serious GI events that could have been immune-mediated colitis; six of these 11 participants (1%) reported the following serious events: ulcerative colitis (*n* = 3), colitis (*n* = 1), Crohn’s disease (*n* = 1), and hemorrhagic enterocolitis (*n* = 1).

#### Malignancies

Potential malignant neoplasms were identified based on a search using the Standardized MedDRA Queries “malignant or unspecified tumors” and medical review. Confirmed malignancies were reported in ten participants (2%): three cases of breast cancer and one case each of basal cell carcinoma, anal cancer, pulmonary carcinoid tumor, clear cell renal cell carcinoma, Hodgkin’s disease, penile cancer, and T-cell lymphoma (later revised to lymphadenopathy, based on the clinical course of the patient’s condition and the results of additional diagnostic testing). Of these cases of malignancy, SAEs of anal cancer, pulmonary carcinoma, and T-cell lymphoma were considered to be related to study treatment by the investigators. Overall, there was no observed pattern in the type of malignancies.

#### Other AEs of special interest

Autoimmune disorders were reported in 7 (2%) of participants: uveitis and autoimmune hepatitis were reported in two participants each; vitiligo, morphoea, rheumatoid arthritis, and Sjögren’s syndrome were reported in one participant each. Serious autoimmune disorders were reported in two (< 1%) participants, and both SAEs were autoimmune hepatitis. One case of serious autoimmune hepatitis was considered by the investigator to be related to study treatment; no action was taken because this participant had been already been withdrawn from the study but was in the 6-month monitoring period after stopping treatment. The second case of autoimmune hepatitis was considered not to be related to study treatment; in this case, treatment was withdrawn and the participant was withdrawn from the study.

Depression and suicidal ideation, identified as AEs of special interest, were reported by 6% of participants. The most commonly reported events in this category were depression (4%) and depressed mood (1%), and the majority of events were mild or moderate in severity. One participant had severe depression and suicidal ideation (two severe events were recorded in the same participant). There were no events of depression and suicidal ideation that led to discontinuation of study treatment. The incidence of depression and suicidal ideation SAEs was < 1%. The SAEs of depression and suicide attempt occurred in the same participant, and both SAEs were assessed as not related to study treatment by the investigator. There were no completed suicides.

There were no confirmed cases of encephalitis or meningoencephalitis observed or retrospectively identified in SELECTED.

### Efficacy

**Fig. 3:**
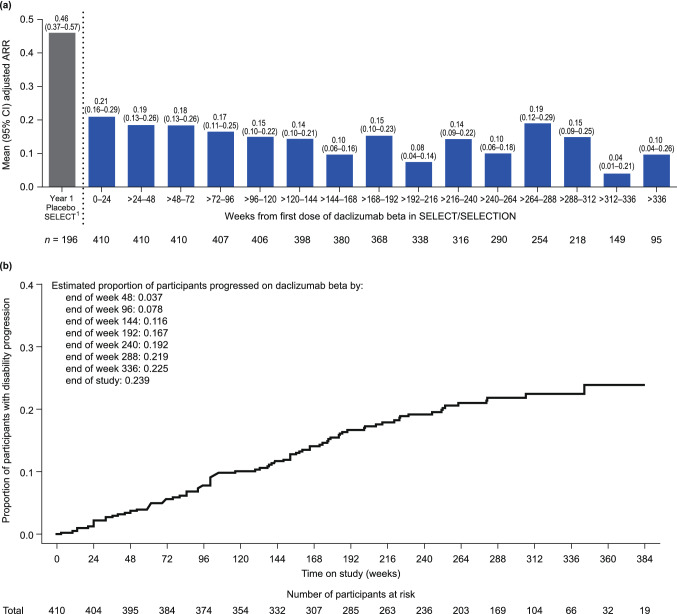
Clinical measures of efficacy in daclizumab beta-treated participants in SELECTED: **a** adjusted ARR by 6-month intervals, **b** 24-week CDP. Efficacy outcomes were assessed from first dose of daclizumab beta received in SELECT or SELECTION. Adjusted ARR was estimated from a Poisson regression adjusted for number of relapses in the year before study entry. Confirmed disability progression is defined as ≥ 1-point increase in EDSS score from a baseline score ≥ 1 or a ≥ 1.5-point increase from a baseline score = 0, sustained for 24 weeks

Efficacy outcomes were assessed based on time from first dose of daclizumab beta received in SELECT or SELECTION. Daclizumab beta efficacy was maintained in participants who continued treatment throughout the study. The adjusted ARR (95% confidence interval [CI]), excluding relapses after alternative MS medication for weeks 0–24, was 0.21 (0.16–0.29), decreasing to 0.10 (0.04–0.26) beyond week 336 (Fig. 3a). The proportion of participants with 24-week CDP remained stable with extended treatment; by the end of SELECTED (up to 8 years of total daclizumab treatment), 23.9% of participants had 24-week CDP (Fig. 3b).

The adjusted mean (95% CI) number of new/newly enlarging T2 hyperintense lesions was 1.95 (1.60–2.37) in year 1 and decreased to 1.58 (0.71–3.52) by year 8 of treatment with daclizumab. The mean (median) annualized PBVC was − 0.77% (− 0.63%) in year 1 and 0.32% (0.41%) by year 8 of treatment with daclizumab beta (see Fig. 1 in Online Resource 1).

## Discussion

The SELECT/SELECTION/SELECTED clinical trials investigated the safety and efficacy of daclizumab beta in more than 400 participants with RRMS. These final findings from the SELECTED extension study indicate that although AEs continued to occur over the extended treatment period, the yearly incidence and severity of AEs remained stable throughout the daclizumab beta treatment period and did not increase with long-term exposure. No new safety concerns were identified in the SELECTED study population. The efficacy benefits seen in a previous 3-year interim analysis of SELECTED [16] were maintained for up to 8 years of treatment.

The overall safety profile of daclizumab beta observed in SELECTED was consistent with that reported in the SELECT and SELECTION studies [12, 15], and in the 3-year, active-control, phase 3 DECIDE study, which compared the safety and efficacy of daclizumab beta with interferon beta-1a in 1841 participants with RRMS [21]. The overall rates of study withdrawal (42%) and treatment discontinuation (45%) were higher for the daclizumab beta 150 mg group in SELECTED than have been observed in extension studies of other MS DMTs [14, 20]. When comparing the approved dosage across trials, the overall rate of discontinuation was 22% in the twice daily delayed-release dimethyl fumarate group in the 5-year interim analysis of the ENDORSE extension study and 12% in the 0.5 mg fingolimod group in the FREEDOMS extension study. In SELECTED, the most common reasons for withdrawal and treatment discontinuation were as a result of AEs or consent withdrawn, and the percentage of participants who discontinued treatment for either of these reasons was higher in SELECTED (22% and 13%) than in ENDORSE (4.5% and 4.9%) or FREEDOMS (2.7% and 6.9%) [14, 20]. However, it is important to note that some discontinuations may have been due to protocol-mandated discontinuation in participants who met threshold ALT/AST elevations, who may or may not have been symptomatic. Although most hepatic AEs were mild or moderate and the majority of participants experiencing hepatic AEs continued treatment in SELECTED, hepatic AEs were the most common cause of treatment discontinuation and withdrawal and included increased ALT, AST, hepatic enzyme, or gamma-glutamyltransferase, and abnormal liver function tests.

Cutaneous AEs were reported in more than one-third of participants over the course of the SELECTED study and are well documented to occur in MS patients treated with daclizumab beta [22, 25]. The incidence reported in SELECTED was similar to that over 2–3 years of daclizumab beta treatment in DECIDE (37%) [21, 22].

The incidence of lymphadenopathy in SELECTED was consistent with the incidences reported previously in DECIDE and across the daclizumab beta clinical trial program (both 6% for data collected as of November 14, 2014) [13, 23]. Most of these events were mild or moderate in severity and resolved while the participant was on daclizumab beta treatment [23]. Additionally, a case of lymphadenopathy was reported in a patient with MS treated with an earlier intravenous form of daclizumab outside of the clinical trial setting [25]. Similar to what has been reported in the clinical trial program, this event resolved following discontinuation of treatment.

Most infectious, cutaneous, and gastrointestinal AEs observed with daclizumab beta were mild or moderate in severity, transient, and generally manageable with standard therapeutic interventions. The annual incidence of these AEs in SELECTED after 8 years of treatment with daclizumab beta were generally similar to those observed in the first and second year of treatment in SELECT and SELECTION [12, 15], respectively, suggesting that up to 8 years of treatment with daclizumab beta did not have a negative cumulative impact on participants with RRMS. Also, an increase in rates of malignancy was not observed with long-term use of daclizumab beta treatment.

Adjusted ARR and the number of new/newly enlarging MRI lesions remained stable and low over up to 8 years of daclizumab beta treatment. Annualized percentage change in whole brain volume decreased over time on daclizumab beta treatment to levels generally in the range observed in healthy controls [5]. The proportion of participants with 24-week CDP remained low, demonstrating that efficacy of daclizumab beta treatment was maintained for up to 8 years of treatment. Overall, the clinical measures of MS disease activity evaluated in SELECTED were consistently lower than those observed in the placebo group in year 1 of SELECT [15].

Long-term extension studies are not without potential limitations. SELECTED was an open-label study without a placebo control group, similar in design to other open-label long-term extension studies [3, 17]. It is important to note that participants in SELECTED did not receive uniform treatment prior to entering the study; participants had been previously randomized to receive either 150 mg or 300 mg daclizumab beta in SELECT, and some may have undergone a 20-week placebo washout period in SELECTION. There were fewer participants with MRI data after year 4. Some participants, but not all, had an MRI scan at week 144 in SELECTED before this assessment was removed in a protocol amendment. This combined with the early transition of participants in SELECTED to EXTEND explains the variation in numbers of participants with MRI results beginning in year 5 of treatment across the SELECTED trilogy. Also, patients doing less well on treatment or who were unresponsive may have elected to stop treatment or not continue in SELECTED, resulting in potential selection bias in favor of those patients who experienced better efficacy or better tolerability with daclizumab beta. Although conclusions regarding efficacy are limited by the lack of a placebo control, the low incidence of relapses and MRI inflammatory lesions over time supports that the treatment effects of daclizumab beta in RRMS can be sustained in the long term.

In SELECTED, most AEs observed with daclizumab beta were mild or moderate in severity and did not increase with long-term exposure. There were no observed cases of inflammatory encephalitis, meningoencephalitis, fulminant liver failure, or fatal liver injury [8, 27]. The efficacy of daclizumab beta on clinical and radiologic MS disease activity outcomes was sustained across yearly treatment intervals for up to 8 years. On March 2, 2018, after SELECTED ended, the sponsors voluntarily withdrew daclizumab beta from the market, given the limited number of patients being treated and the evolving benefit/risk profile; and on March 7, 2018, the EMA suspended marketing authorization and issued a recall of daclizumab beta [1, 7, 10, 28]. The final results from SELECTED contribute to the overall understanding of the effects of long-term daclizumab beta treatment in participants with RRMS in the clinical trial setting and represent the concluding chapter of the SELECT trilogy.

## Electronic supplementary material

Below is the link to the electronic supplementary material.

Supplementary file1 (PDF 199 kb)

## References

[1] Biogen (2018) Biogen and Abbvie announce the voluntary worldwide withdrawal of marketing authorizations for Zinbryta^®^ (daclizumab) for relapsing multiple sclerosis. http://investors.biogen.com/news-releases/news-release-details/biogen-and-abbvie-announce-voluntary-worldwide-withdrawal. Accessed 11 Oct 2019

[2] Cohan S (2016). Therapeutic efficacy of monthly subcutaneous injection of daclizumab in relapsing multiple sclerosis. Biol Targets Ther.

[3] Comi G, O'Connor P, Montalban X, Antel J, Radue EW, Karlsson G, Pohlmann H, Aradhye S, Kappos L (2010). Phase II study of oral fingolimod (FTY720) in multiple sclerosis: 3-year results. Mult Scler.

[4] Confavreux C, Vukusic S (2014). The clinical course of multiple sclerosis. Handb Clin Neurol.

[5] De Stefano N, Stromillo ML, Giorgio A, Bartolozzi ML, Battaglini M, Baldini M, Portaccio E, Amato MP, Sormani MP (2016). Establishing pathological cut-offs of brain atrophy rates in multiple sclerosis. J Neurol Neurosurg Psychiatry.

[6] European Medicines Agency (2017) EMA concludes review of Zinbryta and confirms further restrictions to reduce risk of liver damage. https://www.ema.europa.eu/documents/referral/zinbryta-article-20-referral-ema-concludes-review-zinbryta-confirms-further-restrictions-reduce-risk_en.pdf. Accessed 11 Oct 2019

[7] European Medicines Agency (2018) EMA recommends immediate suspension and recall of multiple sclerosis medicine Zinbryta. http://www.ema.europa.eu/docs/en_GB/document_library/Press_release/2018/03/WC500245167.pdf. Accessed 11 Oct 2019

[8] European Medicines Agency (2018) EMA urgently reviewing multiple sclerosis medicine Zinbryta following cases of inflammatory brain disorders. http://www.ema.europa.eu/docs/en_GB/document_library/Press_release/2018/03/WC500244890.pdf. Accessed 11 Oct 2019

[9] European Medicines Agency (2018) Zinbryta (daclizumab). https://www.ema.europa.eu/en/medicines/human/EPAR/zinbryta. Accessed 11 Oct 2019

[10] Faissner S, Gold R (2018). Efficacy and safety of the newer multiple sclerosis drugs approved since 2010. CNS Drugs.

[11] Giovannoni G, Butzkueven H, Dhib-Jalbut S, Hobart J, Kobelt G, Pepper G, Sormani MP, Thalheim C, Traboulsee A, Vollmer T (2016). Brain health: time matters in multiple sclerosis. Mult Scler Relat Disord.

[12] Giovannoni G, Gold R, Selmaj K, Havrdova E, Montalban X, Radue EW, Stefoski D, McNeill M, Amaravadi L, Sweetser M, Elkins J, O'Neill G (2014). Daclizumab high-yield process in relapsing-remitting multiple sclerosis (SELECTION): a multicentre, randomised, double-blind extension trial. Lancet Neurol.

[13] Giovannoni G, Kappos L, Gold R, Khatri BO, Selmaj K, Umans K, Greenberg SJ, Sweetser M, Elkins J, McCroskery P (2016). Safety and tolerability profile of daclizumab in patients with relapsing-remitting multiple sclerosis: an integrated analysis of clinical studies. Mult Scler Relat Disord.

[14] Gold R, Arnold DL, Bar-Or A, Hutchinson M, Kappos L, Havrdova E, MacManus DG, Yousry TA, Pozzilli C, Selmaj K, Sweetser MT, Zhang R, Yang M, Potts J, Novas M, Miller DH, Kurukulasuriya NC, Fox RJ, Phillips TJ (2017). Long-term effects of delayed-release dimethyl fumarate in multiple sclerosis: Interim analysis of ENDORSE, a randomized extension study. Mult Scler.

[15] Gold R, Giovannoni G, Selmaj K, Havrdova E, Montalban X, Radue EW, Stefoski D, Robinson R, Riester K, Rana J, Elkins J, O'Neill G (2013). Daclizumab high-yield process in relapsing-remitting multiple sclerosis (SELECT): a randomised, double-blind, placebo-controlled trial. Lancet.

[16] Gold R, Radue EW, Giovannoni G, Selmaj K, Havrdova E, Stefoski D, Sprenger T, Montalban X, Cohan S, Umans K, Greenberg SJ, Ozen G, Elkins J (2016). Safety and efficacy of daclizumab in relapsing-remitting multiple sclerosis: 3-year results from the SELECTED open-label extension study. BMC Neurol.

[17] Goodman AD, Bethoux F, Brown TR, Schapiro RT, Cohen R, Marinucci LN, Henney HR, Blight AR (2015). Long-term safety and efficacy of dalfampridine for walking impairment in patients with multiple sclerosis: results of open-label extensions of two phase 3 clinical trials. Mult Scler.

[18] Hart FM, Bainbridge J (2016). Current and emerging treatment of multiple sclerosis. Am J Manag Care.

[19] ICH Expert Working Group (1996) Guideline for good clinical practice E6(R1) ICH harmonised tripartite guideline. www.ich.org/fileadmin/Public_Web_Site/ICH_Products/Guidelines/Efficacy/E6/E6_R1_Guideline.pdf. Accessed 4 Feb 2019

[20] Kappos L, O'Connor P, Radue EW, Polman C, Hohlfeld R, Selmaj K, Ritter S, Schlosshauer R, von Rosenstiel P, Zhang-Auberson L, Francis G (2015). Long-term effects of fingolimod in multiple sclerosis: the randomized FREEDOMS extension trial. Neurology.

[21] Kappos L, Wiendl H, Selmaj K, Arnold DL, Havrdova E, Boyko A, Kaufman M, Rose J, Greenberg S, Sweetser M, Riester K, O'Neill G, Elkins J (2015). Daclizumab HYP versus interferon beta-1a in relapsing multiple sclerosis. N Engl J Med.

[22] Krueger JG, Kircik L, Hougeir F, Friedman A, You X, Lucas N, Greenberg SJ, Sweetser M, Castro-Borrero W, McCroskery P, Elkins J (2016). Cutaneous adverse events in the randomized, double-blind, active-comparator DECIDE study of daclizumab high-yield process versus intramuscular interferon beta-1a in relapsing-remitting multiple sclerosis. Adv Ther.

[23] Lima G, McCroskery P, Dewar R, Castillo JJ, Holman J, Umans K, Fam S (2016). Characterisation of the lymphadenopathy events observed in the daclizumab HYP clinical trials. Mult Scler.

[24] Minocha M, Tran JQ, Sheridan JP, Othman AA (2016). Blockade of the high-affinity interleukin-2 receptors with daclizumab high-yield process: pharmacokinetic/pharmacodynamic analysis of single- and multiple-dose phase I trials. Clin Pharmacokinet.

[25] Oh J, Saidha S, Cortese I, Ohayon J, Bielekova B, Calabresi PA, Newsome SD (2014). Daclizumab-induced adverse events in multiple organ systems in multiple sclerosis. Neurology.

[26] Sheridan JP, Zhang Y, Riester K, Tang MT, Efros L, Shi J, Harris J, Vexler V, Elkins JS (2011). Intermediate-affinity interleukin-2 receptor expression predicts CD56^bright^ natural killer cell expansion after daclizumab treatment in the CHOICE study of patients with multiple sclerosis. Mult Scler.

[27] Stork L, Brück W, von Gottberg P, Pulkowski U, Kirsten F, Glatzel M, Rauer S, Scheibe F, Radbruch H, Hammer E, Stürner KH, Kaulen B, Heesen C, Hoffmann F, Brock S, Pawlitzki M, Bopp T, Metz I (2019). Severe meningo-/encephalitis after daclizumab therapy for multiple sclerosis. Mult Scler.

[28] Lancet T (2018). End of the road for daclizumab in multiple sclerosis. Lancet.

[29] US Food & Drug Administration (2016) FDA approves Zinbryta to treat multiple sclerosis. https://www.fda.gov/news-events/press-announcements/fda-approves-zinbryta-treat-multiple-sclerosis. Accessed 10 Oct 2019

[30] Zhang Y, McClellan M, Efros L, Shi D, Bielekova B, Tang MT, Vexler V, Sheridan JP (2014). Daclizumab reduces CD25 levels on T cells through monocyte-mediated trogocytosis. Mult Scler.

