# Supplementary material for: Long-term safety and efficacy of daclizumab beta in relapsing–remitting multiple sclerosis: 6-year results from the SELECTED open-label extension study
Source: J Neurol. 2020 May 25;267(10):2851–64. doi: 10.1007/s00415-020-09835-y (PMC7501126; doi:10.1007/s00415-020-09835-y)
Supplement: Supplementary file 1 — Supplementary file1 (PDF 199 kb) [file 415_2020_9835_MOESM1_ESM.pdf]

Long-term safety and efficacy of daclizumab beta in relapsing-remitting multiple sclerosis: six-year results from the SELECTED open-label extension study

Ralf Gold[1] · Ernst-Wilhelm Radue[2] · Gavin Giovannoni[3] · Krzysztof Selmaj[4] · Eva Kubala Havrdova[5] · Xavier Montalban[6] · Dusan Stefoski[7] · Till Sprenger[8] · Randy R. Robinson[9] · Sami Fam[10*] · Jonathan Smith[11] · Spyros Chalkias[10] · Giorgio Giannattasio[10] · Gabriel Lima[10*] · Wanda Castro-Borrero[10]

[1]St. Josef-Hospital/Ruhr-University Bochum, Bochum 44791, Germany; [2]Medical Image Analysis Center, University Hospital Basel, Basel, Switzerland; [3]Barts and The London School of Medicine and Dentistry, Queen Mary University of London, London, UK; [4]University of Warmia and Mazury, Department of Neurology, Olsztyn, Poland; [5]First Faculty of Medicine, Department of Neurology, Charles University, Prague, Czech Republic; [6]Hospital Vall d’Hebron University, Barcelona, Spain; [7]Rush University Medical Center, Chicago, IL, USA; [8]DKD Helios Klinik Wiesbaden, Wiesbaden, Germany; [9]AbbVie Inc., Redwood City, CA, USA; [10]Biogen, Cambridge, MA, USA; [11]Biogen, Maidenhead, UK

Correspondence:

Ralf Gold

ralf.gold@rub.de

Online Resource 1

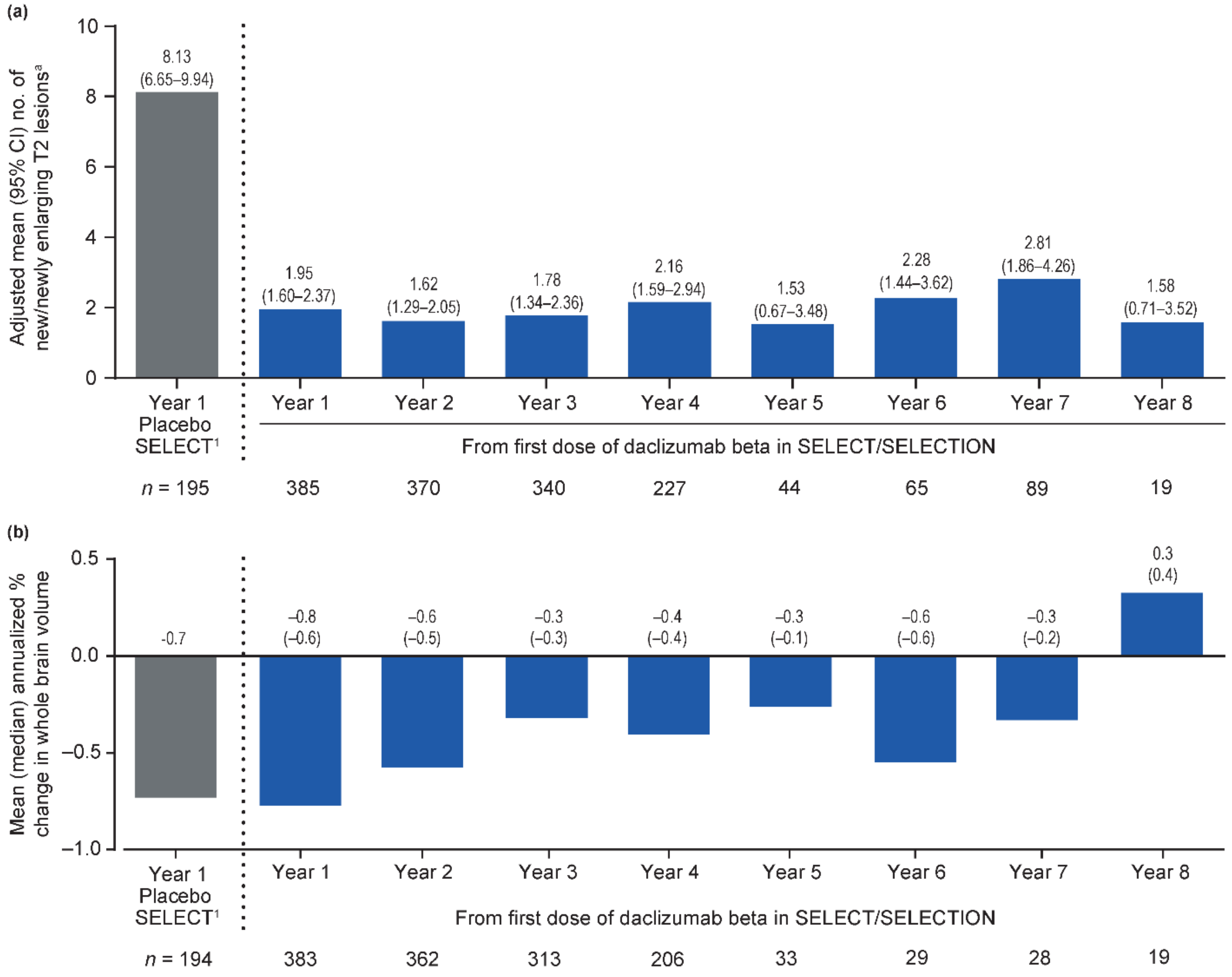

Fig. 1 Radiological measures of efficacy by yearly intervals[b] in daclizumab beta–treated participants in SELECTED: (**a**) number of new/newly enlarging T2 hyperintense lesions[c,d] and (**b**) annualized percentage change in whole brain volume. [a]Estimated from a negative binomial regression adjusted for baseline number of T2 lesions. [b]To be included, the T2 lesion or brain volume parameter must have been reported for the year of assessment and the prior year. Magnetic resonance imaging scans at week 144 in SELECTED were removed from the protocol in May 2013; thus, not all participants have data on these parameters to calculate all yearly intervals across the SELECT trilogy. [c]Adjusted mean was estimated from a negative binomial regression adjusted for baseline number of T2 hyperintense lesions. [d]Adjusted mean number of new/newly enlarging T2 hyperintense lesions since the previous year's magnetic resonance imaging scan
